# Soft optical metamaterials

**DOI:** 10.1186/s40580-020-00226-7

**Published:** 2020-05-26

**Authors:** Yixin Chen, Bin Ai, Zi Jing Wong

**Affiliations:** Department of Aerospace Engineering, Texas A&M University, College Station, Texas, 77843 USA

**Keywords:** Metamaterials, Metasurfaces, Nanophotonics, Soft matter, Nanofabrication, Reconfigurable metamaterials

## Abstract

Optical metamaterials consist of artificially engineered structures exhibiting unprecedented optical properties beyond natural materials. Optical metamaterials offer many novel functionalities, such as super-resolution imaging, negative refraction and invisibility cloaking. However, most optical metamaterials are comprised of rigid materials that lack tunability and flexibility, which hinder their practical applications. This limitation can be overcome by integrating soft matters within the metamaterials or designing responsive metamaterial structures. In addition, soft metamaterials can be reconfigured via optical, electrical, thermal and mechanical stimuli, thus enabling new optical properties and functionalities. This paper reviews different types of soft and reconfigurable optical metamaterials and their fabrication methods, highlighting their exotic properties. Future directions to employ soft optical metamaterials in next-generation metamaterial devices are identified.

## Introduction

Metamaterials consist of artificially engineered sub-wavelength structures that exhibit novel properties [1–6]. Structures such as split ring resonators (SRRs) [7, 8], sub-wavelength wires [9–11], and fishnets [12, 13] can manipulate electromagnetic waves at optical frequencies. These artificial structures can induce electric or magnetic coupling, leading to exotic properties such as negative refractive index [4, 5, 7, 14], perfect absorption [15–18], and hyperbolic dispersion [19, 20]. Over the last two decades, optical metamaterials have enabled possibilities of invisibility cloaking [21, 22], super-resolution imaging [23–25], and efficient energy harvesting [17, 26, 27]. However, the applications of conventional metamaterials are often limited due to their rigid constituent materials, which usually lack flexibility and tuning capability. Besides, they are generally not compatible with biological environments, hindering their potential applications. These issues can be addressed by the incorporation of soft materials, or the sophisticated design of metamaterials that are globally deformable.

It is well accepted that soft materials possess two significant features of complexity and flexibility [28]. The complexity arises from the complex physics behind them, while the flexibility indicates that a mild change in a soft material can lead to a dramatic response [29]. Traditional soft materials include polymers, colloidal dispersions, fluids, and liquid crystals, which are specifically suitable for flexible optoelectronics, biophotonics, and applications in aqueous and biological environments. Moreover, soft materials have several advantages: (i) Properties of soft materials can be easily tuned by external stimuli; (ii) Soft materials are usually low-cost in terms of both raw materials and processing techniques; (iii) Processing of soft materials (e.g. making a metafluid) is often simpler than fabricating a complex rigid metamaterial structure, which typically involve top-down, or high-temperature fabrication techniques. Soft materials offer great opportunities to construct optical metamaterials due to their unique light-matter interaction phenomena [30, 31].

The concept of soft materials can be extended to optical metamaterials [32]. By integrating soft matters within metamaterials or designing globally deformable metamaterials, the flexibility of optical metamaterials are significantly improved. Analogous to soft materials, the properties of soft optical metamaterials are usually adaptive to the environment, and can be easily reconfigured via optical, electrical, thermal or mechanical stimuli. Processing methods of many soft optical metamaterials are simple, low-cost and scalable. Soft optical metamaterials offer cost-effective and versatile opportunities in sensing and dynamic control of optical properties. Soft matters have also been applied in other frequency range, such as infrared [33, 34], acoustic [35, 36], ultrasonic [37, 38], microwave [39–42], and THz frequencies [43–46]. In addition, many soft mechanical metamaterials [47–50] have also been demonstrated. Their exotic mechanical properties were also attained either by integrating soft matters with metamaterial elements or the metamaterials were designed to deform with external stimuli.

Here, we review the properties, fabrication methods, and potential applications of different types of soft metamaterials in the optical frequency regime. Section 2 categorizes optical metamaterials based on their soft material constituents, including liquid crystals, fluids, polymers, and biomaterials. Section 3 discusses the use of electrical, optical, thermal and mechanical stimuli to reconfigure the optical properties of soft optical metamaterials. Finally, Sect. 4 describes common fabrication methods for soft optical metamaterials.

## Soft materials in metamaterials

Most optical metamaterials consist of metallic nanostructure arrays sitting on rigid substrates. The lack of flexibility and shape constraint thus limit metamaterials’ potential applications. One solution is to incorporate soft materials, such as liquid crystals, fluids, biomaterials, and polymers in metamaterials. These soft materials-based metamaterials contain metallic nanostructures to achieve desired plasmonic responses, while soft materials contribute to deformability and tunability. Soft material-based metamaterials offer versatile, tunable, easy processing, and adaptive characteristics for various applications.

### Liquid crystals

**Fig. 1:**
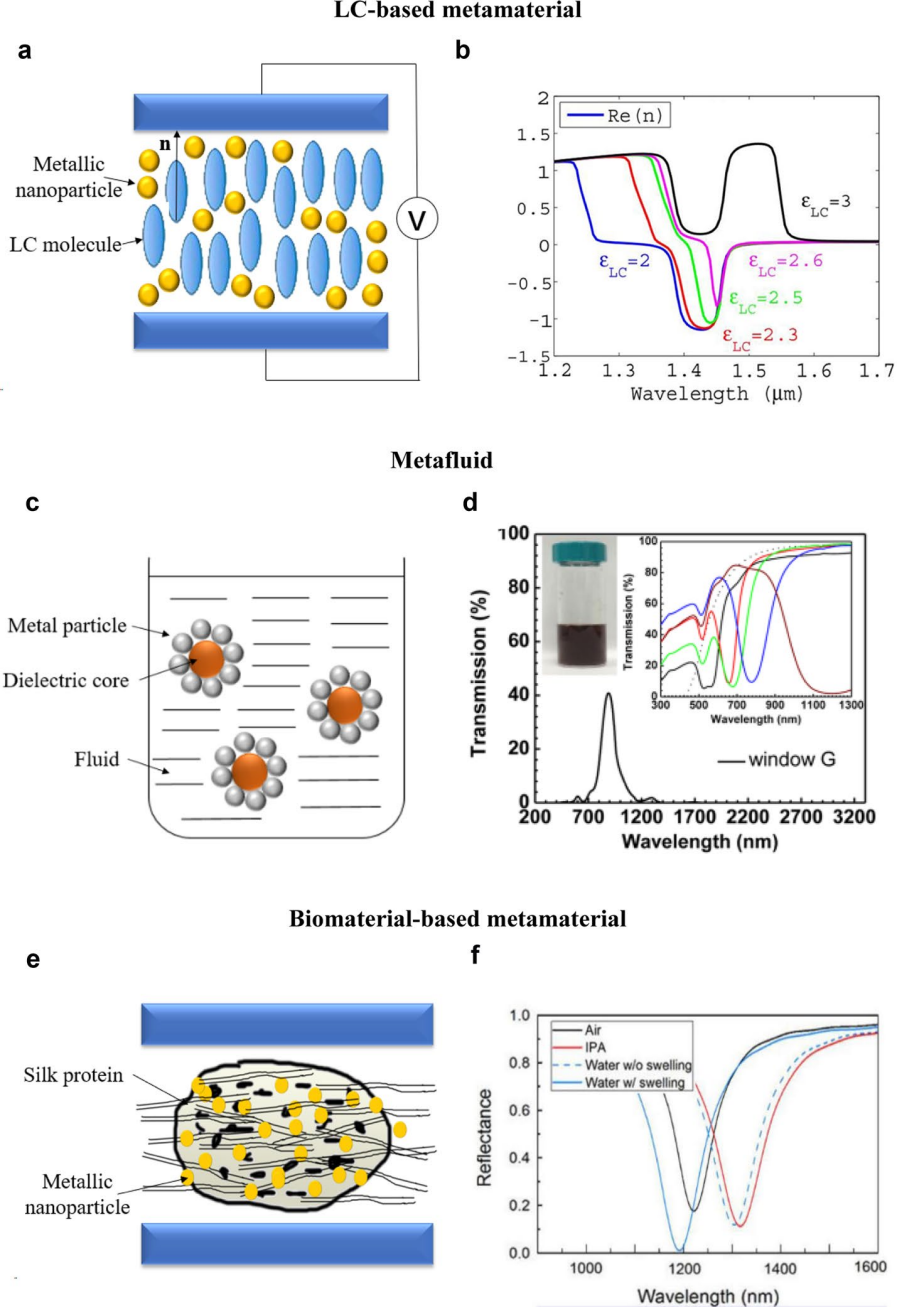
Liquid-crystal (LC) and biomaterials-based metamaterials and metafluids. **a** An illustration of a typical LC-based metamaterial, where metallic structures are dispersed in a LC solution. LC-based metamaterials can achieve optical properties tuning by changing the alignment and orientation of LC molecules via external voltage. **b** The LC-infiltrated metamaterial shows a refractive index ranging from negative to zero and positive values by adjusting the permittivity of LCs. **c** An illustration of metafluid, where metal-dielectric metamolecules are dispersed in a fluid. Metafluid allows the dispersion of different materials or metamolecules to integrate different optical properties in order to attain exotic effective material properties. **d** The transmission spectrum of a metafluid containing boron-doped silicon nanocrystals and five types of Au nanorods (AuNRs) with different geometries. This metafluid possesses a transparency window at 890 nm, with a narrow window width of 0.22 eV. The transparency window is guided by the superposition of the five types of AuNRs whose transmission spectra are shown in the inset. **e** An illustration of a silk protein-based biomaterial metamaterial, where metal nanoparticles are doped in the silk protein structure. **f** The reflection spectra of the bio-compatible silk plasmonic absorber sensor (SPAS) immersed in air (refractive index *n* = 1), isopropyl alcohol (IPA, *n* = 1.37), and water (*n* = 1.32). Larger refractive index can result in reflection resonance redshift in SPAS, facilitating its environment sensing application. **b** Reprinted with permission from [52], Copyright (2007) OSA Publishing. **d** Reprinted with permission from [66], Copyright (2016) ACS Publications. **f** Reprinted with permission from [72], Copyright (2015) ACS Publications

Liquid crystals (LCs) are one of the more common soft materials applied in metamaterials. Metallic nanostructures that possess strong optical response are combined with LCs with changeable crystalline orders. In ordered LCs, the long axes of LC molecules are aligned in the same direction, and this direction vector is called a director (see vector **n** in Fig. 1a). By applying an external electric field, the director orientation can be changed, which modifies its refractive index (RI). The electro-optical response of LC-incorporated metamaterials enables a wide range of RI tuning from negative to zero to positive values. Two device configurations integrating LCs and metamaterial structures were proposed in early years. The first structure consisted of negative-index metamaterial (NIM) unit cells where an anisotropic LC layer was sandwiched between two silver (Ag) stripes [51]. By applying an external electric field, the RI can be reconfigured from − 1.8 to 0 in the near-infrared wavelengths. The second structure used LC cladding layers to cover an Ag NIM [52]. By electrically altering the LC’s permeability from 2 to 6, the RI of this LC-cladded NIM can be tuned from − 1 to + 1.8, as shown in Fig. 1b. Such a large range of RI tuning can hardly be achieved with conventional metamaterials whose properties are fixed upon fabrication.

LC-incorporated metamaterials often utilize LCs in the nematic phase, where the LC molecules are arranged with their long axis parallel to each other without a specific positional order (see the alignment of LC molecules in Fig. 1a). LCs were later incorporated in the fishnet metamaterial structures [53]. Experiments showed that by infiltrating nematic liquid crystals (NLCs) into the air region of fishnet metamaterials, the RI of the combined metamaterial can be tuned from − 2 to + 2 due to the reorientation of LC directors. Besides, NLC-infiltrated fishnet metamaterials can also show a nonlinear optical response, where the nonlinear transmission can be altered with different biased voltages [54].

An alternative way to realize LC-based optical metamaterials is by dispersing metallic nanostructures within the LC. For instance, a nematic LC with dispersed core–shell metallic nanoparticles (NPs) is shown to achieve a tunable RI from − 1 to 1 [55]. A smectic LC-gold nanospheres composite metamaterial is also feasible for tuning optical properties under external voltages [56]. In the smectic phase, LC molecules not only point to the same direction, but they also align with each other to form ‘layers’. Thus, the stability of NP dispersions in smectic LC can be enhanced, and the aggregations of the metallic NPs around the molecules are precluded, rendering it more useful for practical applications [57]. While LC-integrated metamaterials enable exotic optical properties and tuning capability, the interaction between LC molecules and plasmonic nanostructures should be carefully considered in design [58]. Their interaction can diminish the birefringence of LCs and lower the tuning range, a problem that needs to be addressed for practical use of LC-incorporated metamaterials.

### Metafluid

Analogous to the terms “metamaterial” and “metasurface”, metafluid is a fluidic solution containing subwavelength nanostructures to attain unconventional bulk optical properties. The term ‘metafluid’ was first introduced by Urzhumov et al. [59]. In this theoretical work, they proposed to disperse tetrahedrally arranged clusters of gold artificial plasmonic molecules, named tetramer, in a colloidal solution. They showed that the colloidal solution could possess very large, small, or even negative permittivity values. Since then, many efforts have been taken to introduce different nanostructures in colloidal solutions, including gold (Au) and silver nanospheres arranging in tetrahedral, octahedral, icosahedral, and raspberry configurations [60–63]. Kubo et al. developed an n-dodecane solution with dispersed alkanethiol-capped gold NPs [64]. The solution demonstrated a tunable RI in visible and near-infrared wavelengths by controlling the volume fraction of the gold NPs. Such fluidic metamaterials have the advantage of being low-cost and scalable, since solution-based processing is usually easier and cheaper to fabricate than rigid solid-state metamaterials.

In recent years, more studies have been focused on investigating different constituent of metafluids to extend their applications. For example, silver nanoparticles (AgNP) that are packed around polystyrene cores in a fluid solution (as illustrated in Fig. 1c) can exhibit a strong magnetic response [65]. The magnetic dipole scattering from this metafluid is 12% of the strength of electric dipole scattering, and more than one order of magnitude higher than a control sample based on unpacked AgNPs. Negative permeability from − 1 to 0 in the visible region can also be achieved by adjusting the packing ratio. Yang et al. developed a metafluid that has a tunable narrow (~ 100 nm) transparency window within a broadband absorption spanning 200–3300 nm wavelength range, as shown in Fig. 1d [66]. This was made possible by integrating both excitonic (boron-doped silicon nanocrystals, SiNC) and plasmonic materials (gold nanorods, AuNRs) in a colloidal solution. Five types of AuNRs with different geometries combine to produce the narrow transparency window, while the boron-doped SiNC functions as an optical absorber in the ultraviolet and blue-green ranges, and water absorbs in the near-infrared regime. Kim et al. studied the effective refractive index of AuNP metafluids [67]. By considering close-packed and high-volume-factor metamolecules, they discovered that AuNP metafluids could achieve near-zero refractive index and ultrahigh refractive index up to *n*_eff_ = 10 in the visible wavelengths. A colloidal Au nanocube assembly has also shown a high RI of ~ 6.4 at NIR region [68]. In a separate work, Cho et al. developed an all-dielectric optical metafluid using selenium (Se) colloids, where an efficient coupling between its electric and magnetic resonances led to a Kerker-type directional light scattering [69]. The Se colloid metafluid could be used as a generic magnetodielectric building block for fluidic low-loss optical antennas. The same group later synthesized gram-scale and highly-uniform Se metafluids and colloids, which exhibited directional scattering, magnetic resonance, and magnetodielectric bandgaps [70]. Remarkably, colloidal Se metamolecules achieved magnetic resonances with highly uniform nano-sized gaps. In essence, metafluids allow the use of different constituent plasmonic materials to engineer a wide range of optical properties. Such solution-based soft metafluids also break the conventional space and shape constraints, enabling versatile metamaterials with many interesting applications such as adjustable optical filters, optical microfluidic cells, and working fluids in solar-thermal systems.

### Biomaterials

Biomaterials are natural or engineered materials that are compatible with bio-environments. Natural biomaterials from biological tissues and biomacromolecules, such as protein and DNA, are usually soft in texture, transparent, low-cost, and interactive with biological systems. In addition, biomaterials contain sophisticated structures with excellent responsivity, making them useful for biomedical device applications, such as bio-sensing and telemedicine. Recently, biomaterials have also been integrated with optical metamaterials, offering the advantages in terms of cost, tunability, and biocompatibility.

Silk protein is one notable biomaterial with large mechanical tunability and elastomeric properties [71]. Silk protein has been used to create a biocompatible sensing metamaterial, as illustrated in Fig. 1e [72]. The silk plasmonic absorber sensor (SPAS) utilized the local field enhancement (from localized surface plasmon resonance, LSPR) of the metal-silk protein insulator–metal layer, which is analogous to the conventional metal–insulator-metal (MIM) structure. Since silk protein has hydrophilic properties, it exhibits a controllable swelling when immersed in water-alcohol mixtures. The swelling of silk protein leads to resonance shift in the reflection spectrum of the SPAS metamaterial, as shown in Fig. 1f. The amount of resonance shift also depends strongly on the RI of the environment, making SPAS a good RI sensor with a high sensing figure of merit (FOM) up to 1200 nm/RIU (refractive index units).

A hybrid structure consisted of silk protein and AuNP-embedded zinc oxide nanorod arrays also demonstrates superior mechanical flexibility [73]. Remarkably, its optoelectronic properties remain unchanged when it undergoes bending or stretching, making it a robust bio-compatible photodetector. A silk protein MIM structure can also be used as a chemically-tunable optical resonator for pH value sensing [74]. Its resonance is red shifted when swelling, and the swelling can be controlled by environmental stimuli such as the pH value and alcohol concentration.

As biomaterials are active in nature and can facilitate self-assembly of complex structures, biomaterials are also used as building blocks of chiral metamaterials. A DNA-based self-assembly of Au nanoparticles has been demonstrated [75]. The Au nanoparticles were arranged in helices around a DNA origami to form a chiral metamaterial with high precision. The soft assembly enabled chiral responses that were tunable in handedness, color and intensity. Chiral metamaterials composed of DNA origamis attached with arranged plasmonic nanoparticles has been fabricated [76]. By adding or removing phosphate-buffered saline buffers to the environment of the chiral metamaterials, the alignment and orientation of the DNA plasmonic metamolecules could be changed. The changes of DNA alignment and orientation in turn switched the chiral responses of the metamaterial. DNAs also enabled reconfigurable three-dimensional (3D) chiral metamaterials [77]. Here, DNA functioned both as the guide for the self-assembly of the 3D metamaterial, and the building block that enabled the reconfigurability. By adding or removing a strand of DNA, the handedness of the chiral metamaterial could be switched. Amino acid and peptide have also been used to direct the synthesis of chiral AuNPs to fabricate highly twisted nanoparticles [78]. These biomolecule-directed helical nanoparticles display a strong chiral optical activity with a dissymmetry factor of 0.2. A silk fibroin thin film is also employed as a spacer layer to enable tunable circular dichroism (CD) signals in an active plasmonic chiral metamaterial [79]. Bioinspired and biomimetic intramolecular synthesis has been widely employed to fabricate chiral metamaterials [80]. Biomaterial based metamaterials thus provide the opportunities to produce cost-effective, bio-compatible, and mechanically flexible devices such as bio-sensors, soft photodetectors, and chiral molecules.

### Polymers

**Fig. 2:**
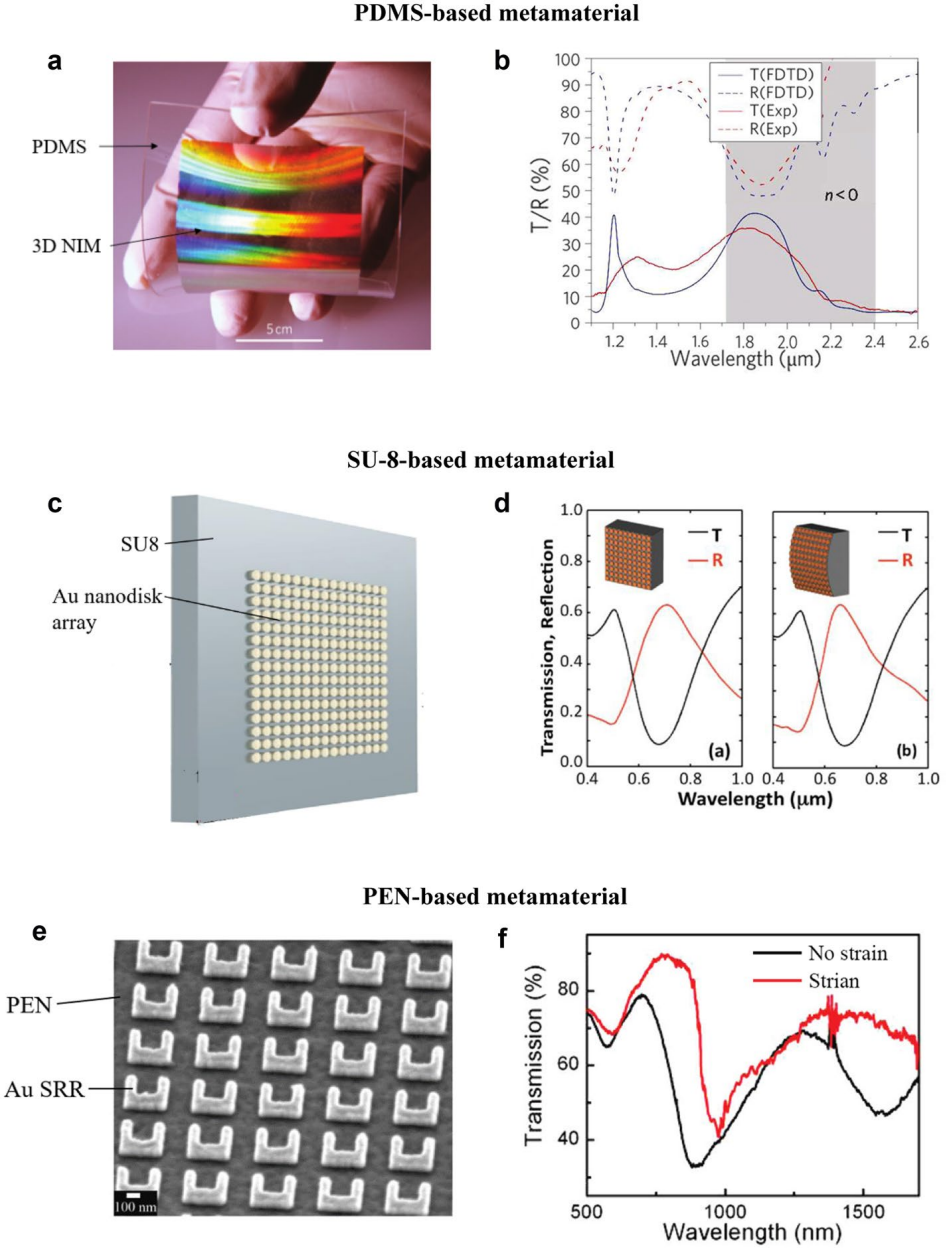
Polymer-based optical metamaterials. **a** A large-area (8.7 cm × 8.7 cm) flexible PDMS based Ag negative index metamaterial (NIM) that shows negative index of refraction at optical frequencies. PDMS has the advantage of enabling direct pattern transfer, benefiting from PDMS’s low surface energy. **b** The transmission (T) and reflection (R) spectra of the large-area PDMS-based Ag NIM metamaterial. The metamaterial shows negative RI at a wavelength range of 1.7 µm to 2.4 µm. **c** An illustration of a flexible metamaterial consists of Au nanodisk array on top of an SU-8 substrate. SU-8 has high chemical and thermal resistance and good mechanical properties, rendering it useful as a flexible metamaterial substrate. **d** The reflection and transmission spectra of a flat (left) and a bent (right) SU-8-based flexible gold nanodisk metamaterial. The reflection is dependent on bending, but transmission of this flexible metamaterial is invariant of bending. **e** A scanning electron microscopy (SEM) image of a PEN-based Au split ring resonator (SRR) metamaterial. PEN has a high glass transition temperature and is transparent in visible and near-infrared (NIR) wavelength ranges. **f** The transmission spectra across visible and NIR wavelength ranges of a PEN-based Au SRR metamaterial with and without an applied out-of-plane strain. Given a strain of 1232 Pa, the electric peak shifts from 894 to 973 nm, showing a sensitivity of 0.06 nm/Pa. **a**, **b** Reprinted with permission from [81], Copyright (2011) Nature Publishing Group. **c**, **d** Reprinted with permission from [89], Copyright (2011) AIP Publishing LLC. **e**, **f** Reprinted with permission from [91], Copyright (2011) ACS Publications

Flexible optical metamaterials are often realized through the use of deformable substrates that are mainly composed of polymers. Polymer-based metamaterials are versatile and low-cost, as they can be easily processed via spin-coating, thermal curing, and well-established microfabrication techniques. Among them, poly-dimethyl-siloxane (PDMS) is a popular choice. As a soft polymer, PDMS can be easily integrated with nonplanar structures and enables direct pattern transfer, benefiting from its low surface energy. The elastic nature of PDMS and its transparency make it a good candidate to realize a deformable metamaterial over a broad optical bandwidth. Chanda et al. fabricated a large-area (8.7 cm × 8.7 cm) bendable NIM on a PDMS polymer substrate, as shown in Fig. 2a [81]. The metamaterial displays negative refractive indices in the NIR wavelength range, as shown in Fig. 2b. Similarly, a nanoimprinted large-area NIM on a PDMS substrate can be engineered to show negative RI in visible wavelength regime [82]. A mechanically tunable titanium dioxide (TiO_2_) dielectric resonator metasurface on a PDMS substrate has also demonstrated strain-dependent transmission spectra [83]. Other PDMS-based optical metamaterials include highly compliant metamaterials with large frequency tunability (~ 400 nm) at NIR range [85], and high-performance surface enhanced Raman scattering (SERS) devices [86].

Polyimide is also widely used as the substrate for metamaterials. Choi et al. demonstrated a flexible metamaterial supported with polyimide layers instead of the more rigid oxide layers [87]. The soft polyimide layers make the fabrication of NIM metamaterial easier, without complicated backside etching. SU-8 is another candidate for the substrate of flexible metamaterials. Falco et al. fabricated nano-antennas and fishnet metamaterials on SU-8 layers, as shown in Fig. 2c [88]. The same group then studied the influence of mechanical deformation on the optical behavior of metamaterials [89, 90]. While some might think that the deformation will always change the properties of metamaterials, they found that the resonant effect is invariant to bending but dependent on the stretching of the flexible metamaterial, as shown in Fig. 2d. Poly(ethylene naphthalate) (PEN) has also been employed as a soft substrate. Xu et al. fabricated a PEN-based Au SRR metamaterial (Fig. 2e) operating at visible and NIR wavelength ranges [91]. Its electrical and magnetic responses are highly sensitive to out-of-plane bending strain, as shown in Fig. 2f. Other than the aforementioned materials, flexible metamaterials have also been fabricated on a polystyrene substrate [92].

Comparing with rigid optical metamaterials, flexible optical metamaterials benefit from their deformability and elasticity, and consequently broaden its functionality in sensing, energy harvesting, large-area cloaking, and beamed light control. For instance, one can tune the resonance frequencies of a PDMS-based SRR metamaterial by varying the stretching force on the metamaterial [85]. Fano resonance frequencies can also be tuned by stretching a metamaterial consisted of Au heptamers on top of a PDMS substrate [93]. Aksu et al. showed that by attaching a layer of highly stretchable low density polyethylene (LDPE) under the PDMS and Au nanorod layers, there could be a resonance peak shift up to 160 nm [94]. Soft polymers enable flexible optical metamaterials with new capabilities, such as negative refractive index [82, 87, 95], nonplanar SERS detectors [86, 92] and molecular sensing [91, 92, 96].

## Reconfigurable soft optical metamaterials

To attain dynamic control and a larger tuning range of the soft metamaterials’ exotic optical properties, soft and active materials are often used. Optical, electrical, thermal and mechanical stimuli are commonly applied on these materials to fully tune their properties, which constitutes a new family of soft metamaterials that is reconfigurable. This provides new degrees of freedom to achieve desired control capabilities such as dynamic tuning, optical switching, and ultrafast electro-optical modulation for optical computing and communication.

### Optically reconfigurable soft metamaterials

**Fig. 3:**
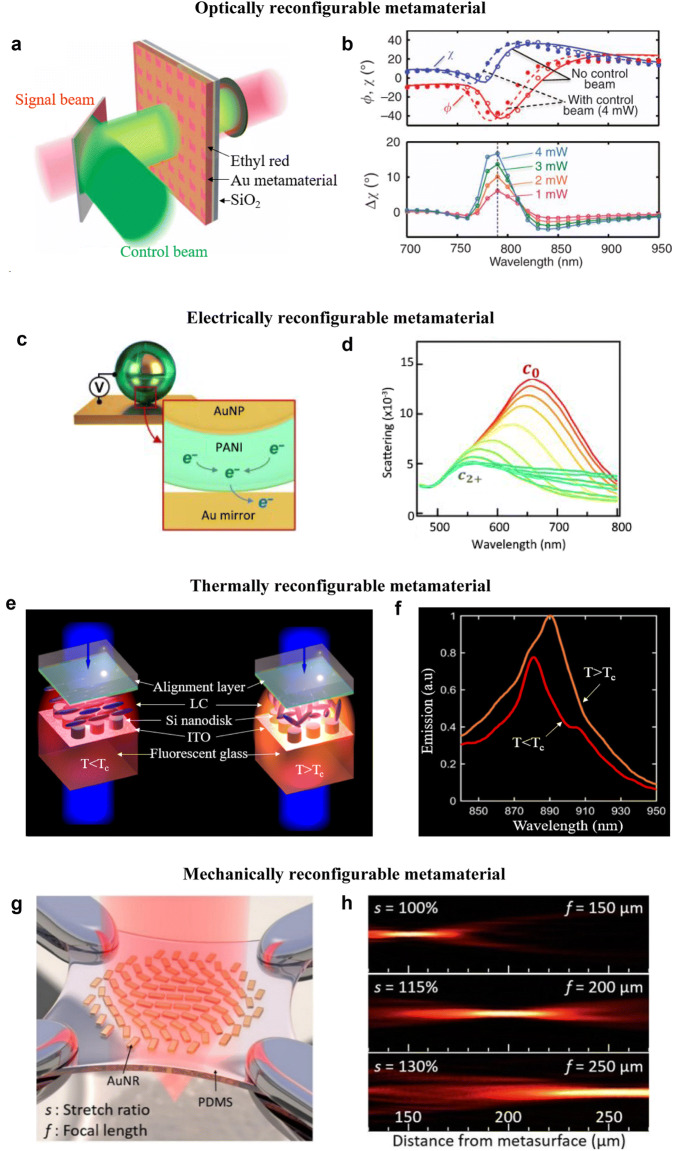
Reconfigurable optical metamaterials triggered by different stimuli. **a** Illustration of an ethyl red based optically reconfigured metamaterial. Upon green laser excitation (control beam), the isomeric state of the ethyl red layer is changed, which results in a refractive index (RI) change and thus switching the optical properties of the soft metamaterial. **b** (Upper panel) Under a 4 mW green light excitation, both polarization azimuth angle ϕ and ellipticity angle *χ* witness blue shifts. (Lower panel) By increasing the control beam intensity, the transmitted ellipticity angle increases at a peak wavelength range of 760–820 nm. **c** Schematic of an electrically reconfigurable polyaniline (PANI) nanoparticle-on-mirror (NPoM) soft metamaterial unit cell. When voltage is applied, the thin PANI layer surrounding the AuNP will undergo a redox process where electrons transfer from PANI to the Au mirror underneath. This leads to a change in RI and the associated optical characteristics. **d** Measured scattering spectra of the scalable PANI NPoM metamaterial. By increasing the voltage from -0.3 V to 0.8 V, the resonance wavelength blue-shifted 79 nm (Δ*λ* = c_0_ − c_2+_) due to the oxidization of PANI coating to PANI^2^. **e** LC-incorporated dielectric metamaterial for thermally tunable spontaneous emission. The device consists of silicon nanodisks embedded in LCs on a fluorescent glass substrate. Below a critical temperature of 58 °C, the LC remains in a nematic phase (left). Upon heating over the critical temperature, the LC changes to an isotropic phase (right) and the RI of LCs varies, enabling thermally tunable spontaneous emission. **f** Spontaneous emission spectra of the metamaterial in (**e**) at different temperatures. Heating above the critical temperature (*T* > *T*_c_) of 58 °C leads to a pronounced red shift of the emission peak. **g** A mechanically reconfigurable metasurface consists of AuNRs on a PDMS substrate with a tunable focal length. **h** Measured longitudinal beam profiles of the AuNR/PDMS metasurface with different stretch ratios. The focal length can be continuously tuned by stretching. **a**, **b** Reprinted with permission from [97], Copyright (2017) Nature Publishing Group. **c**, **d** Reprinted with permission from [110], Copyright (2019) American Association for the Advancement of Science. **e**, **f** Reprinted with permission from [119], Copyright (2018) ACS Publications. **g**, **h** Reprinted with permission from [130], Copyright (2016) ACS Publications

Optically reconfigurable soft metamaterials are typically enabled by active constituents, such as polymers that are highly sensitive to optical excitation. For instance, certain soft polymers undergo a photoisomerization state transition (where the isomers experience structural changes) upon optical illumination. The resulting RI or molecular polarizability changes motivate the use of such polymers as the active layer around the metamaterial structures. Ren et al. reported a reconfigurable metasurface that enabled polarization tuning at optical frequencies by incorporating a photoisomerizable ethyl red layer, as shown in Fig. 3a [97]. The polarization tuning was achieved via optical excitation to switch the coupling condition between the plasmonic modes and the binary isomeric states of the ethyl red layer. The azimuthal angle of the elliptical polarization formed by the metasurface shifted 20° under a moderate switching light power of 4 mW, as shown in Fig. 3b. Azobenzene, a photo-responsive aryl azo compound, has also shown polarization switching capability [98]. Upon irradiation by a linearly polarized light, the isomers would vertically orient with the electric field direction after a series of *trans*–*cis*-*trans* state transitions. When an azobenzene layer was spin-coated on an Au nanohole metamaterial, cross polarization conversion effect is observed. An Au nanocluster metafluid in azobenzene cationic surfactants has demonstrated optically tunable plasmonic responses [99]. By illuminating with ultraviolet light, azobenzene undergoes *trans*–*cis* state transitions. The absorption peak of the AuNP metafluid thus red-shifted by 200 nm with a noticeable metafluid color change. Soft polymer constituents integrated with metamaterial structures enable intense tuning of optical properties under a moderate optical input and facilitate highly compact optical modulating devices as compared to regular tunable metamaterials.

Light can also induce global shape or geometry changes that modify the optical properties of a metamaterial. A good example is the realization of a plasmonic nanomechanical metamaterial consisting of gold stripes and silicon nitride (Si_3_N_4_) layers [100]. Due to the difference in coefficient of thermal expansion (CTE) between the Au and Si_3_N_4_ layers, incident light causes a bimorph deformation that changes the plasmonic resonance, enabling reversible modulation of the metamaterial’s transmissivity. The induced electromagnetic forces between the plasmonic nanostructures also give rise to giant optical nonlinearity. The combined tuning speed and optical nonlinearity compare favorably with other nonlinear optical materials. Without the subwavelength nanostructures and the designed soft system, the nonlinear response would have been moderate and lacks reconfigurability.

### Electrically reconfigurable soft metamaterials

Electrical control of optical properties in metamaterials relies on the voltage-induced changes in their molecular alignment and mechanical structure. Electrically reconfiguring with LCs are based on the realignment of LC molecules under an applied voltage. Early studies showed that by embedding gold nanoparticles [101] or nanorods [102] in LCs or simply placing a LC layer on top of a gold nanorods array [103], the peak of plasmon resonance can be shifted with an external bias, which changes the absorption and transmission spectra. Plasmonic metasurfaces incorporating LCs are also shown to achieve tunable color generation and filters in the visible wavelengths [104]. Electrically-induced reorientation of LC molecules can alter the effective RI of a LC-metasurface, and in turn changes the surface plasmon resonance peak and results in a distinct color tuning. Similarly, LCs are also employed to reconfigure the optical properties of a Mie-resonant all-dielectric metasurface consisted of silicon nanodisks [105]. Recently, an electrically switchable metamaterial color tag based on LCs has shown the capability to continuously control the color (wavelength) of transmitted light under a low applied voltage. 100 nm of wavelength change has been achieved with 0–5 V applied voltage [106]. External voltage can also control the beam bending direction of dielectric metasurfaces when the metasurfaces are incorporated in LCs [107]. An active metasurface spatial light modulator (SLM) with high resolution and efficiency has been achieved. Inclusions of LCs have also enabled electrically tunable color filtering of plasmonic nanohole arrays [108] and visible light modulation of TiO_2_ metasurfaces [109]. These works demonstrate LC-metamaterials’ potentials for high-resolution color printing, holograms, and dynamic displays.

Apart from LCs, several electrochromic soft polymers, whose optical properties can be changed when applying external voltage, have also been used as active media in electrically reconfigurable soft metamaterials. Peng et al. coated a thin layer of electrochromic polyaniline (PANI) on the AuNPs to realize a nanoparticle-on-mirror (NPoM) metamaterial, as shown in Fig. 3c [110]. An external voltage can change PANI’s RI up to Δ*n* = 0.6 due to the oxidization of PANI to PANI^2+^. The scalable soft NPoM metamaterial, demonstrates a continuous color switching of Δ*λ* = 79 nm when the voltage is applied from − 0.3 to 0.8 V, as shown in Fig. 3d. Polystyrene (PS) has also been used as an electrochromic polymer in an Ag MIM nanohole metamaterial [111]. Here, a layer of PS is coated on an Ag-SiO2-Ag nanohole array to function as the active medium for electrical tuning due to the redox process of PS. A surface plasmon resonance (SPR) shift of 72 nm in NIR region has been achieved with applied voltages from 0 to 0.8 V. Electrochromic polymers serving as the active media enable high-sensitivity, large-range reconfiguration of scalable soft optical metamaterials.

Electrically reconfiguring of optical properties can be achieved using the electromechanical effects in a soft metamaterial system, for instance via electrostatic force actuation. Ou et al. showed an electromechanically reconfigurable metamaterial that is made of alternating meander-wire structures on flexible dielectric strings [112]. When a voltage is applied, the meanders and wires will move closer to each other, dramatically changing the transmission and reflection spectrum. This soft metamaterial offers a gigantic electro-optic response (10^−5^–10^−6^ mV^−1^), allowing fast and reversible tuning of optical properties and low-energy, high-contrast non-volatile switching. Another electrically reconfigurable chevron nanowire array metamaterial on an elastic nanomembrane has also been demonstrated [113]. The metamaterial can be reconfigured by the Lorentz force generated from external electric and magnetic fields. The optical property changes as a result of Joule heating and the magneto-electro-optical effect. Reversible transmission tuning has been achieved in this metamaterial, which is promising for nano-tesla level field sensing and magneto-electro-optical modulation. Au meander-wire metamaterials on a flexible PDMS substrate can also be reconfigured by electro-thermo-mechanical effects [114]. By electron injection into the PDMS substrate, the induced heating effect results in the deformation of the PDMS substrate that changes the separation between the meander and the wire. A blue shift of the plasmon resonance frequency is observed at the NIR region. Piezoelectric effects of polymers have also been employed to electrically tune metamaterials. By depositing Ag on a patterned piezoelectric polymer polyvinylidene fluoride (PVDF), the Ag cluster metamaterial would change its shape due to the stretching of the PVDF substrate under external voltage [115]. The Ag cluster soft metamaterial demonstrated an LSPR shift of 100 nm under a moderate electric field intensity of 0.6 V μm^−1^ for 30 s. Arbabi et al. demonstrated a MEMS-tunable silicon metalens with a fast scanning frequency reaching up to kHz range [116]. This tunable metalens has potential in constructing a high field of view with a fast axial scanning capability. Electrical tuning combined with mechanical, thermal and magnetic effects enables fast, reversible and non-volatile reconfiguration of soft optical metamaterials.

### Thermally reconfigurable soft metamaterials

Optical metamaterials have also been reconfigured by thermal stimuli. Thermally reconfigurable metamaterials typically rely on two mechanisms. First, the difference in CTE between two attached constituents in a metamaterial allows the use of temperature to cause deformation, which leads to a change in optical properties. Second, certain materials have temperature-dependent molecules or phases, such as LCs, and shape memory alloys (SMAs). Thermally reconfigurable metamaterials can be achieved by integrating these soft and thermally responsive materials with plasmonic nanostructures. Taking advantage of the materials’ CTE difference, Ou et al. patterned Au slit SRRs on silicon nitride (Si_3_N_4_) to form bilayer membranes that can bend by either heating or cooling [117]. The bending deformation will change the coupling between neighboring plasmonic structures and enable reversible and large-range tuning of the metamaterial’s transmission up to 50%.

LCs can change from an ordered phase to an isotropic phase when the ambient temperature reaches a critical value. This characteristic has been employed to thermally reconfigure metamaterials, as LCs exhibit different optical properties for different phases. Xiao et al. demonstrated a thermally tunable negative permeability optical metamaterial [118]. By covering coupled plasmonic nanostrips with a NLC layer, a magnetic resonance shift from 650 to 632 nm is achieved with the ambient temperature rising from 20 to 50 °C. Similarly, a Mie-resonant dielectric metasurface has been tuned using thermal stimuli, as shown in Fig. 3e [119]. The dielectric metasurface is embedded in an LC cell on a fluorescent glass substrate. By heating up the structure (comprised of a Si nanodisk metasurface and NLCs) over a critical temperature *T*_*c*_ = 58 °C, the spectral response of the metasurface is shifted, and in turn changes the spontaneous emission of the fluorescent substrate, as shown in Fig. 3f. Active thermal tuning and switching of all-dielectric metasurfaces has also been achieved with the use of NLCs [120, 121]. Efficient dynamic switching of laser beam angle from 0° to 12° is achieved when the LC-incorporated metamaterial is heated up to 60 °C [120]. Lewandowski et al. demonstrated a self-assembled AgNP-LC hybrid metamaterial [122]. The AgNP-LC hybrid is aligned in a lamellar pattern at a low temperature (< 70 °C), but upon heating at 95 °C the hybrid becomes isotropically distributed. The metamaterial shows different refractive indices in lamellar and isotropic phases, which enables direct tuning of optical properties such as extinction and transmission.

SMA is a phase change material that can be thermally activated. Having SMAs integrated with metamaterials would facilitate rewritable function, i.e. the ability to configure the metamaterial at its initial state, even after rounds of reconfiguring. Tsuruta et al. presented the first SMA optical metamaterial, whose structure consists of Au etched-in nanostructures sitting on a CuAlNi SMA layer, with a suspended membrane beneath it [123]. When temperature changes, the phase transition of the SMA as well as the CTE difference between the different layers drive the metamaterial to deform. Remarkably, the optical properties of this deformable metamaterial depend not only on the current temperature, but also the temperature history. In [123], the metamaterial is found to be 14% more transparent after cooling to 150 °C, than having been heated to 150 °C. More recently, Nagasaki et al. reported a plasmonic metamaterial based on a gold-coated array of NiTi shape memory nanowires [124]. The hysteretic phase transition (between martensite and austenite states) results in a hysteresis reflection spectrum with 12% difference in reflectivity at the same temperature, depending on whether it has been cooled or heated. Such unique properties cannot be easily attained in conventional metamaterials, rendering SMA-based metamaterials useful for non-volatile switching. The next challenge is to demonstrate even more dramatic shape-changing effect and integration of the SMA with spatially-varying nanostructures to realize functional metasurface optical devices.

### Mechanically reconfigurable soft metamaterials

Mechanical stress and strain can serve as the stimuli to reconfigure the optical properties of metamaterials, especially with the use of soft elastomeric substrates. Huang et al. demonstrated an actively tunable Au dimer plasmonic structure on a elastomeric acrylic substrate [125]. When the metamaterial is being stretched, the surface plasmon response changes accordingly. PDMS is another common soft substrate owing to its elastomeric behavior, low-cost, easy fabrication, and favorable optical properties (such as transparency). Liu et al. fabricated a two-layered Au nanoribbon array on a PDMS substrate and stretched it to tune the surface plasmon resonance [126]. Similarly, an Au heptamers array fabricated on PDMS can have its Fano resonance mechanically tuned [93]. This is attributed to the stress-induced optical modes interaction when the distance between adjacent heptamers (and neighboring structures) are varied. A mechanically reconfigurable all-dielectric metamaterial based on TiO_2_ nanodisk has also been realized on a PDMS substrate [83]. An Au nanorod array embedded in PDMS has also shown reversibly tunable reflection by stretching [127]. Remarkably, the reflection resonance showed ultrasensitive tunability, achieving 48 nm resonance wavelength shift per 1% external strain. Recently, a gold Fano-enhanced metamaterial embedded in PDMS shows reversible CD signals when pressing and releasing the metamaterial with a fiber tip [128].

More recently, researchers explore the use of mechanical stimuli on soft metasurfaces to achieve novel optical functionalities. Tseng et al. developed a new fabrication recipe to pattern and transfer fine aluminum nanostructures on a PDMS substrate and demonstrated a stretchable color display [129]. Remarkably, the displayed color can be tuned to span the entire visible spectrum and produce more vivid colors than the standard red–green–blue (sRGB) color gamut. By introducing spatially-varying Au nanorods (with different orientation to control the phase distribution) on a PDMS substrate, a metasurface lens with a mechanically tunable focal length is demonstrated, as shown in Fig. 3g [130]. The focal length of the metalens can be continuously tuned from 150 µm to 250 µm at 632.8 nm wavelength when the stretching ratio increases from 0 to 30%, as shown in Fig. 3h. Later a dielectric metalens consisted of silicon nanoposts encapsulated in a PDMS layer is also realized, showing an even larger focal length tuning range from 600 µm to 1400 µm at 915 nm wavelength [131]. Malek et al. carefully engineered the position-dependent orientation angle of the AuNRs on a PDMS to build a metasurface hologram that is fully reconfigurable [132]. Three distinctly different holographic images can be switched upon stretching the device. These mechanically reconfigurable metamaterials pave the way for next-generation flexible photonic displays and reconfigurable optical communication devices.

## Fabrication of soft metamaterials

The fast development of soft metamaterials greatly benefits from recent advances in micro/nanofabrication technologies. This is especially so for optical metamaterials, because their component feature size is considerably smaller than microwave and terahertz metamaterials that operate at longer wavelength ranges. The fabrication techniques for soft metamaterials include standard top-down and bottom-up approaches, and non-conventional nanolithography techniques. Herein, we aim to highlight some of representative fabrication processes to realize soft optical metamaterials.

### Conventional top-down approaches

Top-down nanofabrication techniques, such as physical vapor deposition (PVD), chemical vapor deposition (CVD), reactive ion etching (RIE), electron beam lithography (EBL), focused ion beam (FIB) lithography are widely used to fabricate optical metamaterials. Most of the work covered in this review article (from soft material based metamaterials to reconfigurable metamaterials) are realized using these top-down techniques.

**Fig. 4:**
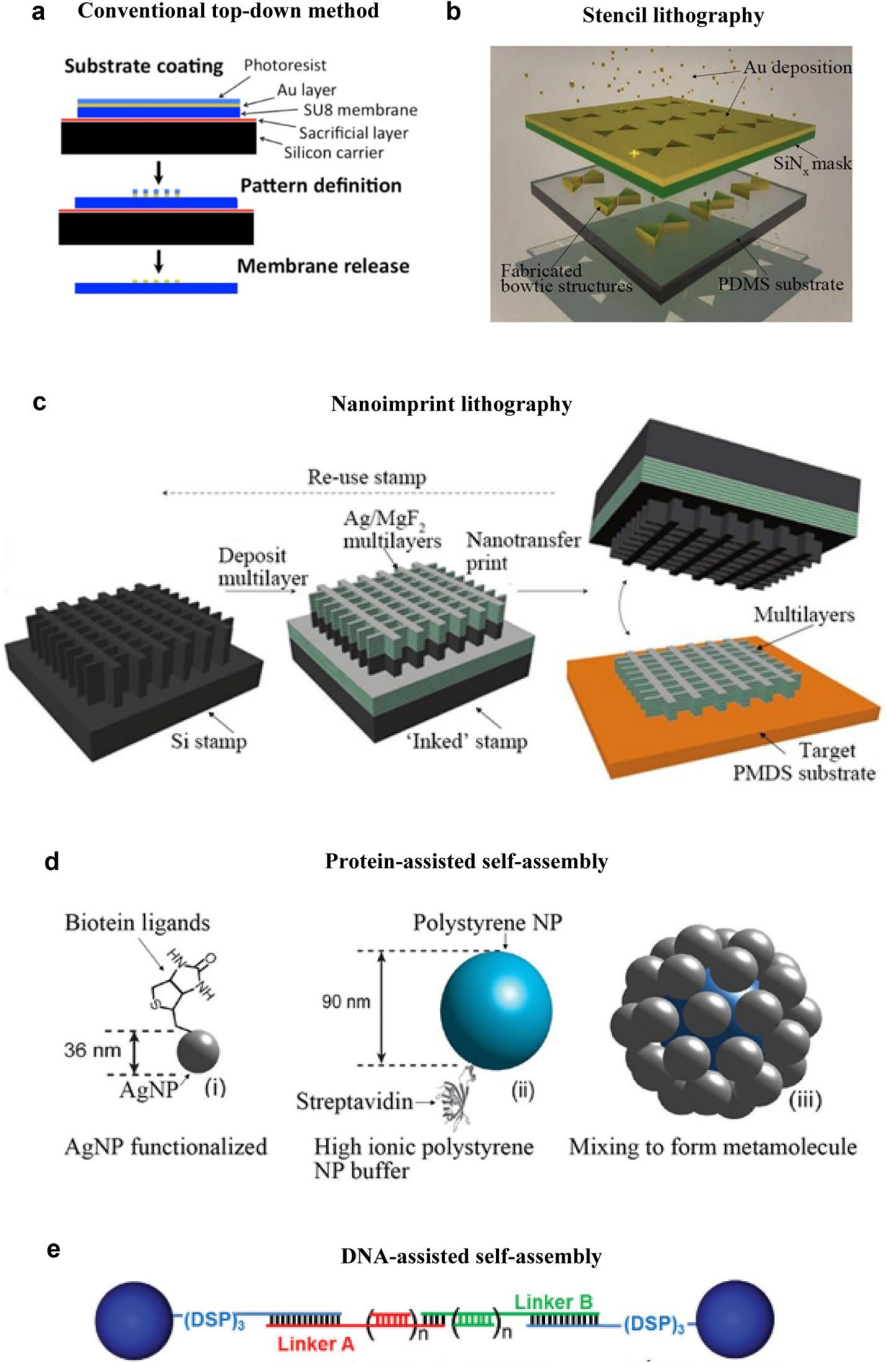
Fabrication methods of soft optical metamaterials. **a** An illustration of the direct top-down patterning of an SU-8 based metamaterial with a sacrificial layer. After the patterning, SU-8-based Au soft metamaterial can be realized by releasing the sacrificial layer. **b** A PDMS-based flexible metamaterial fabricated by stencil lithography. First, a stencil is made and placed above the PDMS substrate with a precise control of the gap distance. Then, gold is deposited through the stencil onto the substrate, forming bowtie nanostructures. **c** Nanoimprint lithography for fabricating a large-area PDMS-based flexible metamaterial. First, a stamp consists of silicon wafers is ‘inked’. Then, the stamp is contacted against a target PDMS substrate. Finally, residual material on the stamp is removed to prepare for re-usage. **d** A protein-assisted self-assembly process to fabricate an AgNP-polystyrene metafluid. AgNPs are first functionalized with biotin terminated ligands (i), and then mixed with a high ionic solution of streptavidin-coated polystyrene NPs (ii) to form a metafluid consisting AgNP-polystyrene metamolecules (iii). Here, the highly specific chemical recognition between protein streptavidin and biotin ligands ensures the AgNPs to be closed packed around the polystyrene NPs. **e** Schematic illustration of DNA-programmable nanoparticle crystallization where the length of the linker strands can be tuned by increasing the value of n. DSP refers to the cyclic dithiol. **a** Reprinted with permission from [89], Copyright (2011) AIP Publishing LLC. **b** Reprinted with permission from [94], Copyright (2011) Wiley–VCH. **c** Reprinted with permission from [81], Copyright (2011) Nature Publishing Group. **d** Reprinted with permission from [65], Copyright (2013) ACS Publications. **e** Reprinted with permission from [143], Copyright (2013) Wiley–VCH

Unlike conventional materials, soft materials or systems are usually deformable, non-conductive, and sensitive to environments (such as high temperature, chemical attack, and mechanical stress), which poses certain challenges to top-down fabrication processes. For example, in EBL, during the release of the soft membrane from the host substrate, lift-off processes suffer from the collapsed and bended membrane. The region of the soft membrane that has already been lifted off will deform and become in contact with the substrate again, blocking more solution from infiltrating for further releasing. To solve the ‘bending membrane’ problem, a sacrificial layer method is a good alternative for releasing the soft metamaterial. Di Falco et al. fabricated a flexible Au metamaterial on a soft SU-8 substrate using EBL, with the assist of a releasing sacrificial layer (XP-SU8), as shown in Fig. 4a [89]. They firstly spun the sacrificial layer onto the host substrate before spinning the soft SU-8 membrane. After the EBL patterning processes, SU-8 based metamaterial is released from the host substrate by immersing in *N*-methylpirrolydone (NMP). Besides, some soft substrates, such as PDMS, suffer from low stability under electron beam, which impedes the direct patterning of nanostructures using EBL. A simple alternative approach is to employ a sacrificial layer-assisted transfer method. Wen et al. firstly spun-coated a Ni sacrificial layer on a stiff SiO_2_ substrate [86]. Standard EBL is then preformed to fabricate Au SRRs on the Ni-coated SiO_2_ substrate. Then, the nanostructure is coated with a soft PDMS layer. With the presence of water, Ni layer can be readily separated from SiO2, only leaving Au SRRs embedded in PDMS. A similar approach was carried out by Kamali et al. for a PDMS-based a-Si flexible metasurface [133]. A germanium sacrificial layer was employed between the PDMS membrane and Si host substrate. After patterning, the soft metasurface is released by immersion in a diluted ammonia solution to dissolve the germanium layer. This simple transfer method involving a sacrificial layer can be effectively applied in the fabrication of soft metamaterials.

On non-conductive substrates, EBL processes suffer from poor structure quality due to the charging effect in the substrates. This problem is detrimental to several polymers based soft metamaterial structures fabricated from EBL. To address the problem, a conducting layer, such as indium tin oxide (ITO) or thin metal, can be deposited underneath the e-beam resist before standard EBL processes. Xu et al. fabricated an Au SRR flexible metamaterial on a non-conductive PEN substrate [91]. Before spin-coating a PMMA e-beam resist on the substrate, they first sputtered an ITO layer to decrease the charging effect. Standard EBL is then conducted to fabricate the flexible metamaterial. In addition, conventional top-down approaches have difficulties in integrating SMAs. Therefore, the fabrication of SMA-based reconfigurable metamaterials usually involves delicate co-sputtering technique and fine tuning of process parameters to achieve the correct composition. Nagasaki et al. carefully fabricated a NiTi SMA nanowire metamaterial [124]. The NiTi layer was deposited by co-sputtering with NiTi and Ni targets. Parameters such as base and working pressures, argon gas flow rate, target-substrate distance, deposition rate were optimized to get the SMA film of the desired thickness and composition.

### Stencil lithography and nanoimprint lithography

Stencil lithography (SL, also known as shadow mask lithography) is another technique commonly employed in the fabrication of soft metamaterials. In SL, a stencil mask is first prepared with etch-through patterns. Then, the stencil mask is used in conjunction with deposition so that only a desired pattern of evaporated material is formed on the substrate. Since there is no direct EBL, exposure on the soft material is eliminated, and charging effects and high energy electron beam-resulted damage can be avoided. Aksu et al. demonstrated a high resolution fabrication method based on SL to create nanopatterns onto flexible substrates, as shown in Fig. 4b [94]. They minimized the stencil-substrate distance and thus minimized diffusion and shadowing problems arising from the gap. The resolution of SL could be as small as 10 nm. Vazquez-Mena et al. successfully fabricated Au and Al nanodots of 20 nm in size by SL on different polymers, including polyimide, SU-8, PDMS and parylene substrates [134]. There are several advantages of using SL for the fabrication of soft metamaterials. First, in SL, thin films can be deposited through the stencil on any planar and nonplanar surfaces. Since soft metamaterials are often employed on nonplanar devices, SL is very suitable for soft metamaterials. Second, the stencil is not in contact with the substrate in SL. Therefore, the lift-off process, which might be problematic for soft substrates, is no longer needed in SL. Besides, since SL is a one-step deposition method, no e-beam resist is needed on the soft substrate, thus avoiding possible material diffusion and contamination issues. However, it is difficult to employ SL for fabricating multi-layer 3D metamaterials, as it requires precise position control of each mask membrane, so that layers can align well with each other. Besides, if the stencil mask is re-used, the hole size will deteriorate, because the deposition of the material can close in at around the edge of the stencil membrane.

Nanoimprinting lithography (NIL) is a replication process using a mold or a stamp to create nanostructures. NIL has the advantages of being low-cost, simple and yet producing high resolution structures [135]. NIL enables high-speed patterning, thus allowing scale up of large-area soft optical metamaterials. Similar to SL, NIL can also be performed on unconventional, non-rigid substrates, which makes NIL particularly suitable for soft metamaterials. Chanda et al. successfully realized a flexible large-area optical NIM using the nanoimprinting method [81]. This nanoimprinting method mainly consists of four steps, as shown in Fig. 4c: (1) Pattern a silicon stamp using soft imprint lithography combined with RIE; (2) ‘inking’ the silicon stamp by electron beam deposition; (3) contacting the stamp against a PDMS substrate to pattern a layer of the desired structure by transferring the deposited material on the elevated regions of the stamp; (4) removing the residual of the deposited materials on the stamp and preparing the stamp for re-usage. In their work, they fabricated 11 layers (one Ag layer on top of five repeats of MgF_2_/Ag layers) on a flexible PDMS substrate. They created many stamps using a single mold, and each stamp was re-used for multiple times. Each unit cell could be printed in a short time of ~ 2.5 s (~ 10^8^ times faster than FIB), making time-efficient manufacturing of large-area flexible metamaterials possible.

Soft lithography is similar to the conventional NIL, but instead of using rigid stamps, it utilizes a soft material, usually PDMS, as the printing stamp. The primary advantage of utilizing soft lithography is that the soft stamp allows an initial contact between the stamp surface and substrate even when the substrate surface is non-planar. This feature provides more flexibility to NIL and the fabrication of soft metamaterials. PDMS is usually chosen as the stamp material, because it is elastomeric, easy to contact and peel off, resistant to many acids, and optically transparent down to wavelength of 300 nm (thus can be used for UV-curing imprint). PDMS soft lithography has been employed to fabricate Ag anode-coated polymer metamaterial solar cells [84]. A PDMS mold, which was previously prepared by a Si master, inscribed desired patterns of recessed areas on the active layer of the solar cell via contact pressing. After removing the PDMS mold, MoO_3_ and Au layers were thermally deposited to the patterns to form a metamaterial solar cell. Large-area 3D soft multilayer NIMs have also been achieved using PDMS soft lithography in conjunction with a subtractive lift-off process [82]. First, a liquid prepolymer was drop-cast on the substrate. The inversed pattern of the NIM is then formed by pressing a prepared PDMS mold against the prepolymer and the subsequent curing to form a solid scaffold. After lifting off the PDMS mold, alternating layers of Ag and MgF_2_ were deposited, forming an NIM on the prepolymer scaffold. The PDMS mold is then removed by a subtractive lift-off process. The NIM metamaterial is then formed after removing the scaffold.

### Bottom-up approaches and solution processing

Soft optical metamaterials significantly benefit from the advancement in solution-based processing and bottom-up self-assembly methods. Self-assembly is not restrained by the limitation of tools and patterning techniques. Instead, self-assembly takes place at the molecular level, enabling the creation of structures at least an order of magnitude smaller than the feature size attainable with top-down methods. Besides, solution or colloid based self-assembly is usually much cheaper and scalable to macroscopic volumes. Furthermore, solution and colloid-based processing is naturally beneficial for integrating multiple constituents to create soft optical metamaterials. Typically, self-assembly processes have to be guided by templates, which can be nanostructures or molecular structures such as liquid crystal, DNA, and protein. The feasibility of realizing isotropic magnetic-based plasmonic effects [136] and plasmonic nanoparticle superlattices as magnetic metamaterials [137] by self-assembly has been studied. Recently, self-assembly is designed to enable colloidal plasmonic superlattices for unnaturally high RI [138], AuNP monolayer as magnetic mirrors for graphene optoelectronics [139], and AuNP dispersion for increasing perovskite solar cell efficiencies [140].

LCs are great candidates as the guide for self-assembly of reconfigurable optical metamaterials because LC molecules can mediate the alignment and aggregation of NPs due to the anisotropy of mesogenic ligands. Gardner et al. showed that by controlling the orientation of the LC molecules, they attained different NPs arrangement and reconfigure the optical properties of the metamaterials formed [141]. On the other hand, a metafluid exhibiting strong optical magnetism was realized by protein-assisted self-assembly colloidal synthesis [65], as shown in Fig. 4d. AgNPs are first functionalized with biotin terminated ligands, and then mixed with a high ionic solution of streptavidin-coated polystyrene NPs to form a metafluid consisting of AgNP-polystyrene metamolecules. Here, the highly specific chemical recognition between protein streptavidin and biotin ligands ensures the AgNPs to be closely packed around the polystyrene NPs. Similarly, a LC-coated AgNP hybrid metamaterial has been fabricated by the ligand exchange reaction [122]. Poly(ethyleneglycol) polymer has been employed as the media to facilitate the self-assembly of silica-core gold-shell metamaterials [142]. Scalable manufacturing of metamaterials can be enabled by self-assembly. A centimeter-scale electrically reconfigurable soft NPoM metamaterial has been fabricated by meniscus-guided nanoparticle assembly [110].

DNA-directed assembly provides a programmable and high-resolution approach for the bottom-up fabrication of metamaterials. DNA-mediated self-assembly is used to synthesize both silver and binary silver-gold nanoparticle superlattices, where they showed epsilon-near-zero (ENZ) responses [143]. Here, DNA is used as a programmable linker to assemble spherical silver NPs into 3D assemblies, as shown in Fig. 4e. Similarly, chiral metamaterials have been fabricated by self-assembly of plasmonic nanoparticles using DNA origami [75]. Remarkably, the plasmonic nanoparticles are arranged in helices with an accuracy smaller than 2 nm. Tailored optical properties can be achieved by adjusting design protocols such as DNA sequences for achieving different arrangement of nanoparticles. Moreover, complex architectures of AuNP clusters have been developed by DNA origami-directed self-assembly [144]. The AuNP clusters network demonstrates strong artificial magnetism such as anti-ferromagnetism, magnetic surface plasmon polaritons, and purely magnetic-based Fano resonance at visible frequencies. 3D DNA origami has been employed to assemble 60–100 nm AuNP metamolecules with high roundness [145]. Programmable DNA origami-directed assembly is used to fabricate micrometer-scale honeycomb 2D lattices as platforms for plasmonic metamaterials [146]. DNA-directed assembly facilitates scalable, programmable, high-resolution, and molecular-level fabrication of plasmonic nanoparticle metamaterials with high roundness and consistency.

Arrays of AuNPs demonstrating broadband high refractive index of 4.2 at visible range have been self-assembled [147]. In this method, hydrophobic AuNPs are first dispersed in a mixture of toluene and hexane. After spreading the mixture on deionized water, the organic solvents are then evaporated in a controllable manner. Due to the differences in evaporation rates of the two organic solvents, AuNP monolayers are formed on the DI water. Remarkably, the assembled arrays of AuNPs can be subsequently transferred to any substrate by hydrographic printing, facilitating the fabrication of non-planar, flexible and tunable metamaterials. Bottom-up self-assembly has also been used to form AuNP clusters with high accuracy, smoothness and roundness with the direction of atomic force microscopy [148, 149], AuNPs embedded in or placed on polymer substrates [150], and a tunable AuNP array with highly precise period and symmetry control [151]. Despite all these works, the understanding of the self-assembly process, especially at the molecular level, is still primitive and warrants further investigation. Another promising method could be the combination of top-down and bottom-up approaches. By fabricating the assembling guides using top-down methods, self-assembly can be achieved at a more controllable level for improving the performance of reconfigurable metamaterials or metafluids. With the development of integrated top-down and bottom-up methods, large-area and scalable production of soft and reconfigurable metamaterials can be envisioned.

## Conclusion and outlook

Over the last two decades, the field of optical metamaterials have witnessed tremendous development in academic research. We believe that the metamaterial community is at a transition point, where more efforts are directed to overcome the challenges to apply metamaterials for practical usage. With the use of external stimuli and soft material constituents, optical metamaterials offer versatile, flexible, and on-demand applications in optoelectronics, imaging, communication, sensing and biophotonics. We believe there is much room for developing new soft optical metamaterials and improving their performances. On the materials side, researchers can identify new soft materials, such as organic fluids, aerogels, and biocompatible materials to integrate with metamaterials. While metasurfaces with spatially-varying phase elements have led to many flat optical devices, their combination with soft materials are still lacking. We thus expect to see continuous development to extend the performance, functionality and reconfigurability of these soft metasurfaces. Besides, the fabrication process can be improved towards cost-effective, reproducible, and scalable production of soft optical metamaterials. Nanofluidics can bring in new opportunities in the synthesis and controlled response of metafluids. Furthermore, programmable or computer-controlled soft metamaterials can be developed to fabricate multifunctional metamaterials with several constituents in a time-efficient manner. It is equally exciting to explore the design of exotic soft metamaterials based on topology, nonlinearity, and hyperbolic dispersion. We foresee that soft optical metamaterials will play an increasingly important role in the fundamental science and technological perspectives.

## Data Availability

Data sharing is not applicable to this article as no datasets were generated or analyzed during the current study.

## Abbreviations

SRR: Split ring resonator
LC: Liquid crystal
RI: Refractive index
NIM: Negative-index metamaterial
NLC: Nematic liquid crystal
NIR: Near-infrared
NP: Nanoparticle
AgNP: Silver nanoparticle
SiNC: Boron-doped silicon nanocrystal
AuNR: Gold nanorod
SPAS: Silk plasmonic absorber sensor
LSPR: Localized surface plasmon resonance
MIM: Metal-insulator–metal
FOM: Figure of merit
CD: Circular dichroism
PDMS: Poly-dimethyl-siloxane
SERS: Surface enhanced Raman scattering
PS: Polystyrene
PEN: Poly(ethylene naphthalate)
LDPE: Low density polyethylene
ITO: Indium tin oxide
PANI: Polyaniline
NPoM: Nanoparticle-on-mirror
SPR: Surface plasmon resonance
PVDF: Polyvinylidene fluoride
CTE: Coefficient of thermal expansion
SLM: Spatial light modulator
ENZ: Epsilon-near-zero
SMA: Shape memory alloy
sRGB: Standard red–green–blue
NMP: *N*-Methylpirrolydone
PVD: Physical vapor deposition
CVD: Chemical vapor deposition
RIE: Reactive ion etching
EBL: Electron beam lithography
FIB: Focused ion beam
SL: Stencil lithography
NIL: Nanoimprinting lithography
